# A Novel Residual Frequency Estimation Method for GNSS Receivers

**DOI:** 10.3390/s18010119

**Published:** 2018-01-04

**Authors:** Tu Thi-Thanh Nguyen, Vinh The La, Tung Hai Ta

**Affiliations:** The International Collaboration Centre for Research and Development on Satellite Navigation Technology in South East Asia (NAVIS), Hanoi University of Science and Technology, Hanoi 100000, Vietnam; tu.nguyenthithanh2@hust.edu.vn (T.T.T.N.); vinh.lathe@hust.edu.vn (V.T.L.)

**Keywords:** GNSS signal processing, frequency estimation, closed-loop tracking, open-loop tracking, differential processing

## Abstract

In Global Navigation Satellite System (GNSS) receivers, residual frequency estimation methods are traditionally applied in the synchronization block to reduce the transient time from acquisition to tracking, or they are used within the frequency estimator to improve its accuracy in open-loop architectures. There are several disadvantages in the current estimation methods, including sensitivity to noise and wide search space size. This paper proposes a new residual frequency estimation method depending on differential processing. Although the complexity of the proposed method is higher than the one of traditional methods, it can lead to more accurate estimates, without increasing the size of the search space.

## 1. Introduction

In Global Navigation Satellite System (GNSS) receivers, the synchronization block is one of the most important elements. It synchronizes the received signal and demodulates the navigation message, which is necessary to compute the Position, Velocity, and Time (PVT). Traditionally, this block is divided into two parts: acquisition and tracking. The aim of the acquisition stage is to determine which are the satellites in view, and to estimate their code delay θ, and Doppler frequency $f_{d}$. These raw parameters are then used to initialize the next stage, in which the satellite signals are tracked.

The acquisition can be done computing the correlation between the received signal and a series of several tentative signals generated by the receiver. The tentative signal with the highest correlation value, provided that this is higher than a predefined threshold, is assumed to be equal to the received signal. In the tracking stage, a Delay Lock Loop (DLL) is used to refine the code delays, whereas the frequencies can be refined by a Frequency Lock Loop (FLL) and/or a Phase Lock Loop (PLL). The lock loops include a discriminator to estimate the residual in the frequency and/or phase of the tentative signal w.r.t that of the received signal, and a filter is then used to reduce the noise in the residual. Then, this residual is fed back to the numerical controlled oscillator (NCO) [1]. Although PLL brings a higher accuracy than FLL, it can work well only if the residual of the initial frequency is small enough. However, the accuracy of the frequency estimation provided by the acquisition stage is such that the residual is in the range of a few hundred Hertz, which is too large an input value for the proper behavior of the PLL. Therefore, it is necessary to adopt a frequency estimation refinement method before PLL using an FLL [1], or refinements based on the results of the acquisition stage [2].

In PLL and FLL, the output measurements are fed back to re-configure the lock loops; therefore, they are considered as closed-loops. The most significant advantage of such closed-loops is their memory and their computational resource minimization [3]. However, closed-loops are vulnerable to fading effects and cycle slips [4,5]. Moreover, the possibility to extend integration time to improve the sensitivity of closed-loops in harsh environments is limited by the stability issue and the dynamics of signals [5,6,7]. Open-loop solutions are another option to overcome the previously mentioned limitations. In open-loop approach, not only one single signal, but batches of local signals are used, and the carrier frequency is estimated by a frequency estimator. By this way, the signal observability is improved. The full observation in time domain, frequency domain, and join time-frequency domain allows for estimating the carrier frequency in high dynamic environments. In addition, since a large search space is used, the tracking process can be recovered immediately after a temporary signal loss [3]. However, in current GNSS receivers, open-loop solutions are not as popular as close-loop ones because of their high computational cost.

It can be seen that the frequency estimator plays an important role in the synchronization block of a GNSS receiver. In literature, several estimators have been introduced, such as [2,4,8,9,10,11,12,13], etc. They can be divided into two different groups. The first one relies on the phase difference between a burst of consecutive and non-consecutive correlation values, and the second one is based on the Fast Fourier Transform (FFT).

For the first group, Kay’s method [8] is one of the most well-known methods. In it, the residual frequency is calculated from the phase difference between two consecutive correlation values. Several modified versions of Kay’s method were proposed to improve the estimation performance. The generalized Kay’s method [4] improves the accuracy of estimation in high dynamic environments. In [9], Tahir et al. introduced two modifications of Kay’s method. The first one is based on the wrapped phase algorithm while the second tries to detect and correct the error of the estimation of Kay’s method. However, these methods can provide good results only in high signal-to-noise ratio conditions. Another approach that depends on the different phase of both consecutive and non-consecutive correlation values, can be found in [2,14,15]. If only consecutive correlation values are used, all of them converge to the conventional differential estimator in [16]. These methods can work well only if the residual amount is rather small. On the contrary, when the residual frequency exceeds a threshold, which depends on the maximum distance between non-consecutive values in a combination, the estimate that these methods can provide is worse than the one in [16]. To effectively use these methods, the frequency search space must be adequately large.

Recently, the second group of estimators, which based on FFT, has gain more interests [10,11,12,13]. To reduce the computational load, on some of such estimators, the FFT algorithm is implemented for the integration results instead of IF signal samples [10,12,13]. However, because of the trade-off between the frequency resolution and the computational complexity, the maximum error of frequency estimation depends on the number of FFT points. In [12], several ways are introduced to improve the accuracy of the FFT-based estimators, such as: sinc interpolation, zero-forcing, and double FFT. The performance of these proposals are good in both team of accuracy and complexity.

This paper introduces a new method to estimate the residual frequency, which belongs to the first group of estimators, and can be considered as an improved version of the method introduced in [2]. This new method has better performances than other existing methods in the same group. Similar to other methods, it uses the phase difference between both consecutive and non-consecutive correlation values to bring a more accurate estimation of the residual frequency. However, the difference is that its accuracy can be maintained without enlarging the frequency search space.

The paper is organized as follows: Section 2 gives an overview of the residual frequency estimation problem and summarizes some typical solutions; Section 3 describes the proposed method; then, the performance of the proposed solution is presented in Section 5 and compared with existing methods. Finally, conclusions and future work are provided in Section 6.

## 2. Residual Carrier Frequency Problem

### 2.1. Signal Model

The digitalized GNSS signal r[n] available in a receiver, after the Analog-to-Digital Converter (ADC) block, can be represented as:

(1) $$r\left[n\right]=\sqrt{P}d\left[n+\theta \right]c\left[n+\theta \right]\cos\left(2\pi \left(f_{IF}+f_{d}\right)nT_{s}+\varphi \right)+n_{w}\left[n\right]$$

where *P* is the power of the signal; d[n] denotes the navigation data symbol; c[n] is the PRN code at a time instance *n*; $n_{w}\left[n\right]$ is additive white Gaussian noise (AWGN) with zero mean (μ=0), and variance $\sigma_{n}^{2}$; $f_{IF}$ and $f_{d}$ denote the Intermediate Frequency (Hz) and the Doppler shift (Hz), respectively; $T_{s}$ is the sampling period (s), which is the inverse of the sampling frequency $F_{s}$; θ is the received code delay (sample); and φ is the initial phase of the received signal (radiant).

The correlation value between the received signal r[n] and the tentative signal $\hat{r}\left[n\right]$ can be expressed as [2]:

(2) $$\begin{array}{ccc} R_{m} & = & \sum_{n=(m-1)N+1}^{mN}\left\{r\left[n\right]\hat{r}\left[n\right]\right\}\\ & = & \sum_{n=(m-1)N}^{mN}\left\{r\left[n\right]\hat{c}\left[n+\hat{\theta }\right]e^{j(2\pi \left(f_{IF}+{\hat{f}}_{d_{m}}\right)nT_{s})}\right\}\\ & ≜ & s_{m}+w_{m} \end{array}$$

where *m* stands for the index of the coherent integration interval [(m−1)N,mN], $N=\lfloor T_{coh}F_{s}\rfloor$ denotes the coherent integration time $T_{coh}$ (s) in samples; $s_{m},w_{m}$ are the signal and the noise components, respectively. Assuming that the frequency is constant in a small interval, it can be written:

(3) $$\left\{\begin{array}{ccc} s_{m} & \approx & \frac{N}{2}\sqrt{P}d\left[n+\theta \right]R\left[\tau \right]\mathrm{sinc}\left(▵{\bar{f}}_{d}T_{coh}\right)e^{j(2\pi m▵{\bar{f}}_{d}T_{coh}+\varphi )}\\ & ≜ & Ge^{j\Phi_{m}}\\ w_{m} & = & \sum_{n=(k-1)N}^{kN}n_{W}\left[n\right]\hat{c}\left[n+\hat{\theta }\right]e^{j\left[2\pi \left(f_{IF}+{\hat{f}}_{d}\right)nT_{S}\right]} \end{array}\right.$$

where ($\tau =\theta -\hat{\theta }$) and ($▵{\bar{f}}_{d}=f_{d}-{\hat{f}}_{d}$) are the average difference between actual and tentative code delays and Doppler shifts during an interval; and R[τ] is the autocorrelation function [17]. $w_{m}$ can be considered as a normal distribution random variable with zero mean and variance $\sigma_{w}^{2}=N\sigma_{n}^{2}$ ($w_{m}∼N\left(0,N\sigma_{n}^{2}\right)$).

As pointed out in [2], there are three factors that cause loss of measured correlation value: the bit transition, the residual code in the autocorrelation function R[τ], and the residual frequency. The solution of residual code and data transition can be found in [18,19]. Moreover, some modern GNSS signals introduce pilot channels, which do not contain data bits. Although the pilot channel still has a secondary code, it can be synchronized by multi-layer code based acquisition [20]. Therefore, the data transition problem can be ignored, and, for simplicity, in the following sections, code and data are considered to be wiped off (τ=0 and d[n+θ]=1). The residual frequency can be eliminated by using Double Differentially (DDF) Coherent method [21], or estimated by the frequency estimators as introduced in Section 1. The DDF method works effectively for the signal detection; however, the frequency estimation is still needed for the tracking process. In this paper, only the problem of estimating $▵{\bar{f}}_{d}$ is considered. In reality, the Doppler frequency is not constant but changes continuously overtime. This causes a drift component in the residual frequencies as analyzed in [22]. The Doppler drift also affects the correlation value and the accuracy of the frequency estimate. However, in the case of not high dynamic receiver, the Doppler drift can be ignored [22]. Moreover, the results in [7] showed that, in the high dynamic case, the convention frequency estimator can follow the Doppler changes with a proper architecture of tracking loop. Therefore, the Doppler drift is not considered in this paper.

### 2.2. Frequency Estimation Methods

From Equations (2) and (3), it can be seen that, in the free-noise case, the phase difference between two consecutive correlation intervals is equal to $2\pi ▵{\bar{f}}_{d}T_{coh}$:

(4) $$\begin{array}{ccc} s_{m}s_{m-1}^{*} & = & G^{2}e^{\Phi_{m}-\Phi_{m-1}}\\ & = & G^{2}e^{j2\pi ▵{\bar{f}}_{d}T_{coh}} \end{array}$$

Based on Equation (4), in order to estimate the residual frequency, several methods have been proposed in the literature and are hereafter shortly described.

#### 2.2.1. Classical Kay’s Method

**Figure 1:**
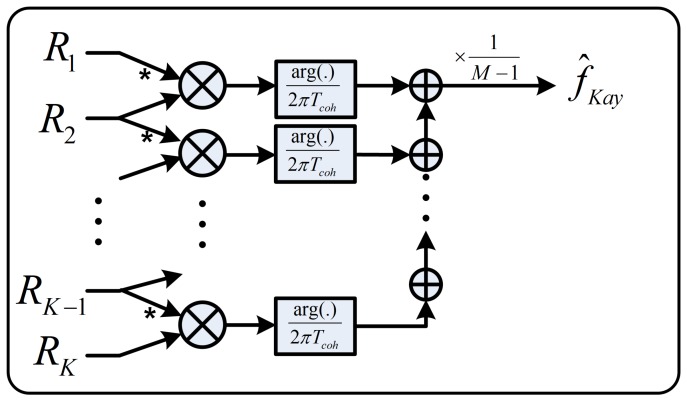
Classical Kay’s methods.

The method, which was introduced in [8], estimates the residual frequency from the phase difference between two consecutive correlation values (see Figure 1). The estimator has the form:

(5) $$\begin{array}{ccc} ▵{\hat{f}}_{Kay} & = & \frac{1}{2\pi T_{coh}\left(M-1\right)}\sum_{m=2}^{M}\arg\left(R_{m}R_{m-1}^{*}\right) \end{array}$$

where the total observation time is $T_{0}=M\cdot T_{coh}$ (seconds), and *M* is the number of observed interval.

This method is based on the phase wrapping algorithm. It has a low computational complexity, but it only works effectively with high SNR because it is sensitive to noise [9].

#### 2.2.2. Conventional Differential Combination Method

In [16], a method to estimate the frequency that is the residual in the closest tentative signal was proposed. This method, referred to Conventional Differential Combination (CDC) method, was proved to be suitable for signals with a very low SNR. The estimated residual frequency is derived as:

(6) $$\begin{array}{ccc} ▵{\hat{f}}_{CDC} & = & \frac{\arg\left(\sum_{m=2}^{M}R_{m}R_{m-1}^{*}\right)}{2\pi T_{coh}} \end{array}$$

**Figure 2:**
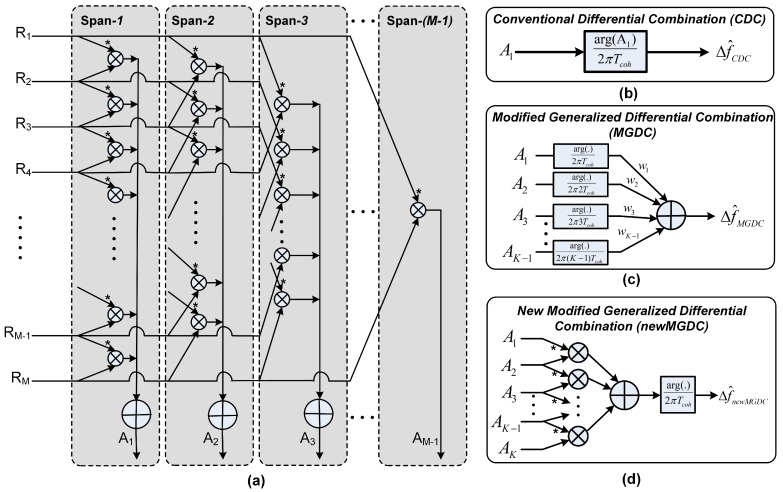
Differential processing for frequency estimating. (**a**) differential operations; (**b**) conventional differential combination; (**c**) modified generalized differential combination; (**d**) modified generalized differential combination.

The difference between the CDC method and Kay’s method is the order of argument function and integration (see Figure 1 and Figure 2b). It should be noted that the argument function is a nonlinear function, while the integration acts as a linear noise filter. Therefore, if the argument function is performed before the integration, the effect of white noise in the correlation values will change, and the filtering effectiveness of the integration will be decreased.

#### 2.2.3. Modified Generalized Differential Combination Method

In [2], a new method based on post-correlation combination is proposed and used to improve the sensitivity of acquisition stage. It is also called the Modified Generalized Differential Combination (MGDC) method. In this method, the correlator outputs are differently combined in different spans, as depicted in Figure 2a,c. A span-*i*, which is denoted as $A_{i}$, is defined as the differential combination of the correlator outputs evenly spaced *i* intervals:

(7) $$A_{i}=\sum_{m=i+1}^{M}R_{m}R_{m-i}^{*}$$

where 1≤i≤M−1.

Considering the free-noise case, substituting the contribution of the useful signal in Equation (3) into Equation (7), it can be rewritten:

(8) $$\begin{array}{ccc} A_{i} & = & \sum_{m=i+1}^{M}G^{2}e^{j2\pi i▵{\bar{f}}_{d}T_{coh}}\\ & = & \left(M-i\right)G^{2}e^{j2\pi i▵{\bar{f}}_{d}T_{coh}} \end{array}$$

where $G=\sqrt{2P}R\left[\tau \right]\mathrm{sinc}\left(▵{\bar{f}}_{d}T_{coh}\right)$.

Therefore, the residual frequency can be estimated by:

(9) $$\begin{array}{ccc} ▵{\bar{f}}_{d,\mathrm{span}-i} & = & \frac{1}{i}\frac{\arg\left(A_{i}\right)}{2\pi T_{coh}}\\ ▵{\hat{f}}_{MGDC} & = & \sum_{i=1}^{K}w_{i}▵{\bar{f}}_{d,\mathrm{span}-i} \end{array}$$

where $w_{i}$ is chosen depends on the number of combinations in the respective span.

The number of usable spans for the MGDC method is limited as presented in the next section. However, the results in [2] shows that: if the number of usable span is more than one, the more number of valid spans is used, the better performance of the estimation is because the more information is explored.

### 2.3. Wrapped Phase Problem

In all the methods above, the argument function is used to estimate the residual phase component in the correlation values. It should be noted that the argument function returns a wrapped phase, which means:

(10) $$\begin{array}{cc} \arg\left(Ce^{2k\pi +\phi }\right)=\phi ;\; & \forall \phi \in [-\pi ,\pi ],\forall k\in Z \end{array}$$

Thus, if the actual phase is unwrapped (k≠0), the estimation phase error can be multiple times of 2π. To avoid the problem, the phase at the input of argument function has to be in the range [−π,π].

Before considering wrapped phase problem for each method above, it is important to note that, if the search space is defined as the parallel code phase search acquisition [23], we have:

(11) $$\begin{array}{c} \left\{\begin{array}{c} -\frac{\Delta f_{Step}}{2}\leq ▵{\bar{f}}_{d}\leq \frac{\Delta f_{Step}}{2}\\ \Delta f_{Step}\leq \frac{1}{2T_{coh}} \end{array}\right.\\ \Rightarrow -\frac{1}{4T_{coh}}\leq ▵{\bar{f}}_{d}\leq \frac{1}{4T_{coh}} \end{array}$$

where $\Delta f_{Step}$ is the Doppler step size of the search space.

Inequality (11) presents the variation interval of the residual frequency before the estimation. For Kay’s method and the CDC method, from Equations (4)–(6), the wrapped phase constraint can be written as:

(12) $$\begin{array}{cc} & -\pi \leq 2\pi ▵{\bar{f}}_{d}T_{coh}\leq \pi \\ \Leftrightarrow & \frac{-1}{2T_{coh}}\leq ▵{\bar{f}}_{d}\leq \frac{1}{2T_{coh}} \end{array}$$

Since Inequality (11) is achieved before the frequency estimation, Inequality (12) is always satisfied. For the MGDC method, the wrapped phase constrain can be written as:

(13) $$\begin{array}{cc} & -\pi \leq \arg(A_{i})\leq \pi \\ \Leftrightarrow & -\pi \leq 2\pi i▵{\bar{f}}_{d}T_{coh}\leq \pi \\ \Leftrightarrow & \frac{-1}{2T_{coh}}\leq i▵{\bar{f}}_{d}\leq \frac{1}{2T_{coh}} \end{array}$$

**Figure 3:**
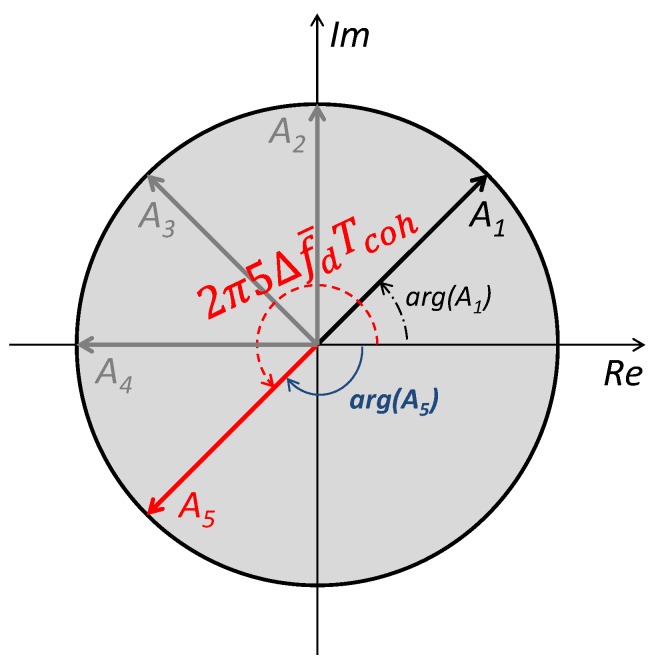
Limit of usable spans in Modified Generalized Differential Combination (MGDC) methods.

Consequently, the maximum number of spans in Equation (9) that can be used is constrained. For examples, if $T_{coh}=1$ ms, and $▵{\bar{f}}_{d}=125$ Hz, from Inequality (13), i≤4, and the combination $A_{5}$ must not be used to estimate the residual frequency. The wrapped and unwrapped phase of vector $A_{5}$ are different as shown in Figure 3. The function $\arg(A_{5})$ does not return the unwrapped phase value (dashed angle in Figure 3), and instead it provides the wrapped phase (solid angle in Figure 3), so that including vector $A_{5}$ in Equation (9) leads to a consistent error in the estimated phase.

From Inequalities (11) and (13), in the worst case—when the Doppler step is maximum—the maximum number of usable spans is 2. However, it is not recommended to satisfy sharp relation (13) since noise has unexpected influence on the computation of the argument function because, in such conditions, the noise could lead to consistent errors in the phase determination. It follows that only span-1 should be used on this case that, obviously, is equivalent to the CDC method. The only ways to increase the number of usable spans is to reduce the Doppler step in the search space. However, this leads to an increase in complexity, especially when a long integration time is used.

## 3. Proposed Method

Recall that, in the MGDC method, higher order spans returning a wrapped phase different from the unwrapped one (as it is the case of span-5 in Figure 3) can not be used for the phase estimation according to Equation (9). To overcome this problem, note that, in the ideal case, the phase difference between two consecutive span-*i* vectors is equal to $2\pi ▵{\bar{f}}_{d}T_{coh}$ regardless of the index of the spans:

(14) $$\begin{array}{cc} A_{1,i} & =A_{i}A_{i-1}^{*}\;\;\left(2\leq i\leq M-1\right)\\ & =\left(\left(M-i\right)G^{2}e^{j2\pi i▵{\bar{f}}_{d}T_{coh}}\right)\\ & \;\times \left(\left(M-i-1\right)G^{2}e^{j2\pi \left(i-1\right)▵{\bar{f}}_{d}T_{coh}}\right)\\ & =\left(M-i\right)\left(M-i-1\right)G^{4}e^{j2\pi ▵{\bar{f}}_{d}T_{coh}}\\ & ≜G_{i}e^{j2\pi ▵{\bar{f}}_{d}T_{coh}} \end{array}$$

where $G_{i}=G^{4}\left(M-i\right)\left(M-i-1\right)$. The index of used span no longer appears in Equation (14). This allows for estimating the residual phase using $A_{1,i}$ and without being affected by the unwrapped phase problem.

$A_{i}$ can be considered as the first order differential of correlations, and $A_{1,i}$ can be considered as a second order differential. From Equation (8), for a specified number of correlation values, the higher span order would use less number of correlation values, which leads to smaller magnitude of $A_{i}$ and $A_{i}$. In other words, the magnitude $G_{i}$ represents the amount of information in each combination, and thus can be considered as a weighted value in a new frequency estimator (see Figure 2a,d), which follows:

(15) $$\begin{array}{c} ▵{\hat{f}}_{newMGDC}=\frac{\arg\left(\sum_{i=1}^{K}A_{i}A_{i-1}^{*}\right)}{2\pi T_{coh}} \end{array}$$

where 2≤K≤M−1 is the number of combinations. A large value of *M* is not recommended because it causes a great delay in the tracking process and reduces the accuracy instantaneously in the case of high dynamic users. Therefore, in this paper, *M* is chosen as equal to or less than 200.

Since the unwrapped phase problem is avoided, the number of combinations in this estimation method is not limited as it is in the MGDC method. Hence, this method is more effective than the others in case of a large residual phase, and does not require an increasing of the frequency domain size in the search space. Especially in weak signal scenarios, where adopting the longer $T_{coh}$ is a convenient approach to increase the sensitivity of the synchronization block, smaller Doppler step leads to a noticeable increase in the acquisition/batch processor complexity, which will be shown in the next section.

Another important point about the derivation of relation (15) is the order in which argument and sum functions are computed that can affect obtained results, as it has been already discussed in Section 2.2.2. The authors have first implemented the sum that, acting as a linear filter, reduces the effect of the noise before the nonlinear argument function is computed.

## 4. Theoretical Analysis

This section presents the probability density function of estimated frequency of CDC and MGDC methods. To the best of the author’s knowledge, the theoretical analysis for the Kay’s method and new MGDC method are not easy to derive. However, their performance can be assessed through the Monte Carlo simulation, as introduced in Section 5.

According to [24], a complex random variable *X* with the real part ℜ(X) and image part ℑ(X) are independent and follow the normal distribution: $ℜ\left(X\right)∼N\left(\mu_{I},\sigma_{I}^{2}\right)$ and $ℜ\left(X\right)∼N\left(\mu_{Q},\sigma_{Q}^{2}\right)$, has joint probability density function (pdf):

(16) $$p\left(ℜ\left(X\right),ℑ\left(X\right)\right)=\frac{1}{2\pi \sigma_{I}\sigma_{Q}}e^{-\frac{{\left(ℜ\left(X\right)-\mu_{I}\right)}^{2}}{\sigma_{I}^{2}}-\frac{{\left(ℑ\left(X\right)-\mu_{Q}\right)}^{2}}{\sigma_{Q}^{2}}}$$

By using the polar coordinates, (16) is transformed into:

(17) $$\begin{array}{c} p\left(r,\phi \right)=\frac{r}{2\pi \sigma_{I}\sigma_{Q}}e^{-\frac{{\left(r\cos\left(\phi \right)-\mu_{I}\right)}^{2}}{\sigma_{I}^{2}}-\frac{{\left(r\sin\left(\phi \right)-\mu_{Q}\right)}^{2}}{\sigma_{Q}^{2}}} \end{array}$$

where: r=|X|,ϕ=arg(X), and ϕ∈[−π,π].

Therefore, by integrating (17) over *r*, we obtain the pdf of ϕ as follows [16]:

(18) $$\begin{array}{ccc} p_{\phi }\left(\phi \right) & = & \int_{0}^{\infty }p\left(r,\phi \right)dr\\ & = & \frac{\gamma }{2\alpha^{2}\left(\phi \right)}+\frac{\gamma \beta \left(\phi \right)\sqrt{\pi }}{4\alpha^{3}\left(\phi \right)}e^{\frac{\beta^{2}\left(\phi \right)}{4\alpha^{2}\left(\phi \right)}}\mathrm{erfc}\left(-\frac{\beta (\phi )}{2\alpha (\phi )}\right) \end{array}$$

where:

(19) $$\begin{array}{ccc} \gamma & ≜ & \frac{e^{\left(-\frac{\mu_{I}^{2}}{2\sigma_{I}^{2}}-\frac{\mu_{Q}^{2}}{2\sigma_{Q}^{2}}\right)}}{2\pi \sigma_{I}\sigma_{Q}} \end{array}$$

(20) $$\begin{array}{ccc} \alpha (\phi ) & ≜ & \sqrt{\frac{\cos^{2}\left(\phi \right)}{2\sigma_{I}^{2}}+\frac{\sin^{2}\left(\phi \right)}{2\sigma_{Q}^{2}}} \end{array}$$

(21) $$\begin{array}{ccc} \beta (\phi ) & ≜ & \frac{\mu_{I}\cos\left(\phi \right)}{\sigma_{I}^{2}}+\frac{\mu_{Q}\sin\left(\phi \right)}{\sigma_{Q}^{2}} \end{array}$$

Substituting $\phi =2\pi T_{coh}▵\hat{f}$ into Equation (18), we have:

(22) $$\begin{array}{ccc} p_{▵\hat{f}}\left(▵\hat{f}\right) & = & p_{\phi }\left(2\pi T_{coh}▵\hat{f}\right) \end{array}$$

Equation (22) can be applied to compute the probability of frequency estimated from the complex normal random variable *X*. However, applying Equation (22) for Kay’s method is inappropriate because $R_{m}R_{m-1}^{*}$ is a product of two normal random variables, which does not follow the normal distribution. The pdf of $R_{m}R_{m-1}^{*}$ is quite complex as computed in [25], and it is not easy to derive $p_{▵\hat{f}}\left(▵\hat{f}\right)$ from that. For CDC and MGDC methods, if the number of combination *M* is high enough (M≥20), applying the Centre Limit Theorem (CLT), $A_{i}$ in Equation (7) can be considered as following the normal distribution (for i=1, MGDC is equivalent to CDC). Therefore, Equation (22) can be used to obtain the estimated frequency distribution of CDC and MGDC methods.

Applying Equation (22), the expected values and variances of the real and the image part of $A_{i}$ are needed. From Equations (3) and (7), we have:

(23) $$\begin{array}{cc} A_{i} & =\sum_{m=i+1}^{M}R_{m}R_{m-i}^{*}\\ & =\sum_{m=i+1}^{M}{\left(s_{k-1}+w_{k-1}\right)}^{*}\left(s_{k}+w_{k}\right)\\ & =\left(\sum_{m=i+1}^{M}s_{k}s_{k-1}^{*}\right)+\left(\sum_{m=i+1}^{M}s_{k}w_{k-1}^{*}+w_{k}s_{k-1}^{*}\right)+\left(\sum_{m=i+1}^{M}w_{k}w_{k-1}^{*}\right)\\ & ≜A_{i,1}+A_{i,2}+A_{i,3} \end{array}$$

From Equation (3), $s_{k}$ and $s_{k-1}$ are deterministic, so:

(24) $$A_{i,1}=\sum_{m=i+1}^{M}s_{k}s_{k-1}^{*}=\left(M-i\right)G^{2}e^{j2\pi i\Delta \hat{f}T_{coh}}$$

As proved in [22]:

(25) $$\sigma_{A_{i,3}}^{2}=Var\left(\sum_{m=i+1}^{M}w_{k}w_{k-1}^{*}\right)=\left(M-i\right)\sigma_{w}^{4}$$

$A_{i,2}$ can be represented as:

(26) $$\begin{array}{cc} A_{i,2} & =\sum_{m=i+1}^{M}s_{k}w_{k-1}^{*}+w_{k}s_{k-1}^{*}\\ & =\sum_{m=i+1}^{2i}s_{k}w_{k-1}^{*}+\sum_{m=i+1}^{M-i}w_{k}\left(s_{k-i}+s_{k+1}\right)+\sum_{m=M-i+1}^{M}w_{k}^{*}s_{k-1}\\ & ≜A_{i,21}+A_{i,22}+A_{i,23} \end{array}$$

The components $A_{i,21}$ and $A_{i,23}$ can be analysed by the same way, as follows:

(27) $$\begin{array}{cc} \sigma_{A_{i,21}^{I}}^{2} & =E\left(\sum_{m=i+1}^{2i}{\left[G(\cos\left(\phi_{k}\right)w_{I}+\sin\left(\phi_{k}\right)w_{Q})\right]}^{2}\right)\\ & =\frac{i}{2}G^{2}\sigma_{w}^{2}. \end{array}$$

Then:

(28) $$\begin{array}{c} \sigma_{A_{i,21}^{I}}^{2}=\sigma_{A_{i,21}^{Q}}^{2}=\sigma_{A_{i,23}^{I}}^{2}=\sigma_{A_{i,23}^{Q}}^{2}=\frac{i}{2}G^{2}\sigma_{w}^{2} \end{array}$$

The in-phase component of $A_{i,22}$ can be computed as:

(29) $$\begin{array}{cc} \sigma_{A_{i,22}^{I}}^{2} & =E\left(\sum_{m=i+1}^{M-i}G^{2}{\left[\left(\cos\left(\phi_{k+1}\right)+\cos\left(\phi_{k-1}\right)\right)w_{I}+\left(\sin\left(\phi_{k+1}\right)+\sin\left(\phi_{k-1}\right)\right)w_{Q}\right]}^{2}\right)\\ & =\frac{1}{2}G^{2}\sigma_{w}^{2}\sum_{m=i+1}^{M-i}\left[{\left(\cos\left(\phi_{k+1}\right)+\cos\left(\phi_{k-1}\right)\right)}^{2}+{\left(\sin\left(\phi_{k+1}\right)+\sin\left(\phi_{k-1}\right)\right)}^{2}\right]\\ & =\frac{1}{2}G^{2}\sigma_{w}^{2}\sum_{m=i+1}^{M-i}\left[2+2\cos(\phi_{k+1}-\phi_{k-1})\right]\\ & =2\left(M-2i\right)G^{2}\sigma_{w}^{2}\cos^{2}\left(2\pi i▵{\bar{f}}_{d}\right) \end{array}$$

Similarly, the quadrature component of $A_{i,22}$ is:

(30) $$\begin{array}{cc} \sigma_{A_{i,22}^{I}}^{2} & =2\left(M-2i\right)G^{2}\sigma_{w}^{2}\sin^{2}\left(2\pi i▵{\bar{f}}_{d}\right) \end{array}$$

Consequently, the variance of the real part and image part of $A_{i}$ can be written as:

(31) $$\begin{array}{cc} \sigma_{ℜ(A_{i})}^{2} & =iG^{2}\sigma_{w}^{2}+2\left(M-2i\right)G^{2}\sigma_{w}^{2}\cos^{2}\left(2\pi i▵{\bar{f}}_{d}\right)+\frac{M-i}{2}\sigma_{w}^{4}\\ \sigma_{ℑ(A_{i})}^{2} & =iG^{2}\sigma_{w}^{2}+2\left(M-2i\right)G^{2}\sigma_{w}^{2}\sin^{2}\left(2\pi i▵{\bar{f}}_{d}\right)+\frac{M-i}{2}\sigma_{w}^{4} \end{array}$$

From Equation (24), the mean values of the real part and image part of $A_{i}$ are:

(32) $$\begin{array}{cc} \mu_{ℜ(A_{i})} & =ℜ\left(A_{i,1}\right)=\left(M-i\right)G^{2}\cos\left(2\pi i\Delta \hat{f}T_{coh}\right)\\ \mu_{ℑ(A_{i})} & =ℑ\left(A_{i,1}\right)=\left(M-i\right)G^{2}\sin\left(2\pi i\Delta \hat{f}T_{coh}\right) \end{array}$$

For the CDC method, applying Equations (31) and (32) with i=1 into Equation (22), the pdf of the frequency estimated by CDC method is obtained. Then, the mean of the $▵{\hat{f}}_{CDC}$ can be computed as:

(33) $$E\left(▵{\hat{f}}_{CDC}\right)=\int_{-\frac{1}{2\pi T_{coh}}}^{\frac{1}{2\pi T_{coh}}}f\times p_{\phi ,CDC}\left(2\pi T_{coh}f\right)df$$

and the variance of the estimated frequency is:

(34) $$\mathrm{Var}\left(▵{\hat{f}}_{CDC}\right)=\int_{-\frac{1}{2\pi T_{coh}}}^{\frac{1}{2\pi T_{coh}}}f^{2}\times p_{\phi ,CDC}\left(2\pi T_{coh}f\right)df-E\left(▵{\hat{f}}_{CDC}\right)$$

**Figure 4:**
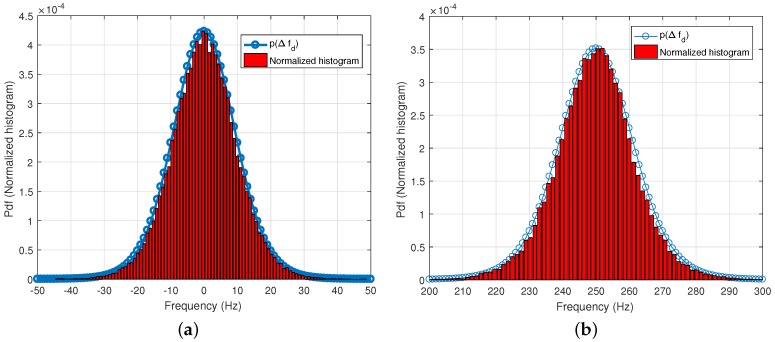
The probability density function of estimated frequency by the Conventional Differential Combination (CDC) method versus histograms (obtained by 500,000 simulation runs) (**a**) $▵\bar{f}=0$ Hz; (**b**) $▵\bar{f}=250$ Hz.

Figure 4 shows the theoretical line of $p_{▵\hat{f},CDC}\left(▵\hat{f}\right)$, and the normalized histogram of $▵{\hat{f}}_{CDC}$ obtained by Monte Carlo simulation with 500,000 simulation runs. The input residual $▵\bar{f}=0$ Hz in Figure 4a, and $▵\bar{f}=250$ Hz in Figure 4b. The matching between pdfs and the histograms validates the theoretical analysis above.

For the MGDC method, because the combinations $A_{i}$ are correlated, it is not easy to compute the pdf and variance of estimated frequency. However, the pdf of the estimated frequency at each span can be calculated by applying Equations (31) and (32) into Equation (22), with *i* being the index of the considered span. Then, the mean value of $▵{\hat{f}}_{MGDC}$ can be calculated as:

(35) $$E\left(▵{\hat{f}}_{MGDC}\right)=\sum_{i=1}^{K}w_{i}\int_{-\frac{1}{2\pi T_{coh}}}^{\frac{1}{2\pi T_{coh}}}f\times p_{\phi ,MGDCspan-i}\left(2\pi T_{coh}f\right)df$$

## 5. Performance Analysis

In this section, the accuracy and the complexity of the proposed method is assessed and compared with the methods introduced in Section 2.2 using Monte Carlo simulation for 50,000 test points. Regarding the accuracy of the evaluated estimators, two signal conditions are considered. The first one is the nominal range of $C/N_{0}$, which is 35 dB-Hz and above [18,26], and the second one is the weak signals, in which $C/N_{0}$ is in the range of [20÷35] dB-Hz. In the nominal conditions, the integration time equals 1 ms and the number of combinations M=20 are adequate to detect and track the signal [7]. While the signal is weak, two possible solutions can be adopted: extending the integration time, and/or increasing the number of combinations. In this section, to guarantee the proper complexity, the used number of combinations *M* does not exceed 200, the number of combinations for the second order differential is K=20, and the integration time is extended up to 10 ms. The residual frequencies are simulated with considering Inequality (11).

### 5.1. The Accuracy of Estimations

**Figure 5:**
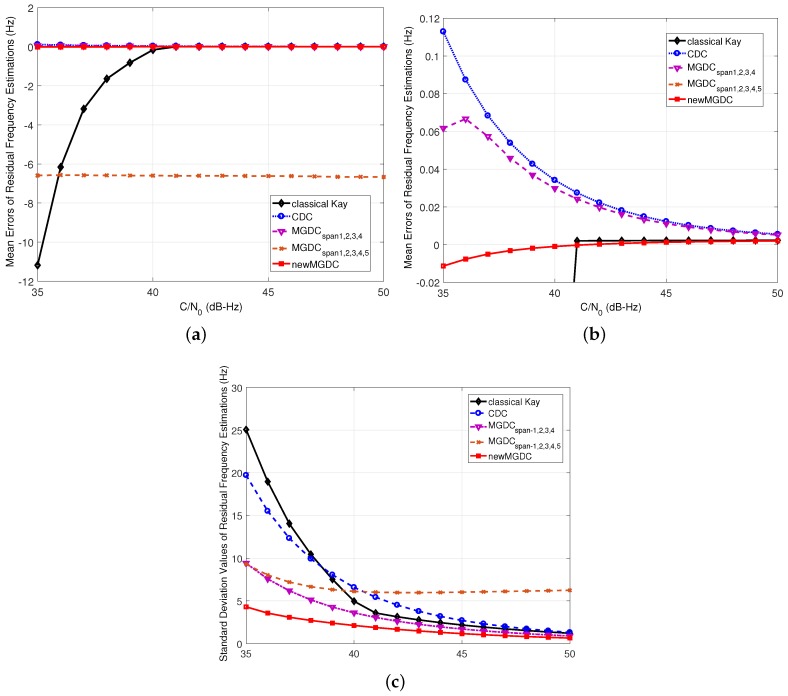
Performance of new residual frequency estimation method ($\Delta {\bar{f}}_{d}=100\mathrm{Hz},T_{coh}=1\mathrm{ms},$ M=20, K=19). (**a**) mean errors of estimations; (**b**) zoomed view of (**a**); (**c**) standard deviation of estimations.

**Figure 6:**
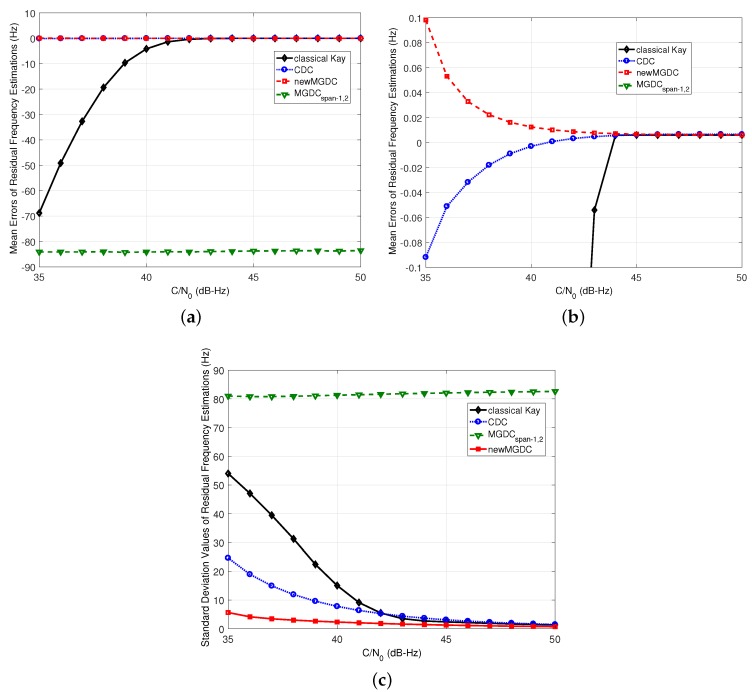
Performance of new residual frequency estimation method ($\Delta {\bar{f}}_{d}=250\mathrm{Hz},T_{coh}=1\mathrm{ms},$ M=20, K=19). (**a**) mean errors of estimations; (**b**) zoomed view of (**a**); (**c**) standard deviation of estimations.

Figure 5 shows the simulation results of mean errors and standard deviation estimations of the methods when the residual frequency $▵{\bar{f}}_{d}=100$ Hz and $T_{coh}=1$ ms. From Inequality (13), i≤5, or the number of usable spans in MGDC method is up to 5. However, the simulation results in both mean errors and standard deviation estimations of MGDC span-1,2,3,4 show a better performance than MGDC span-1,2,3,4,5. This is because when the equal sign occurs in Inequality (13), the idea vector $A_{5}$ is matched with the boundary between −π and π. This leads to an ambiguity in the estimated phase of vector $A_{5}$, which can jump from −π to π under noise effect, and causes errors in the estimation. The same result can be found in Figure 6, which shows the results of worst case, when $▵{\bar{f}}_{d}=\frac{1}{4T_{coh}}=250$ Hz. Although the theoretical analysis in Section 2.2 indicates that the maximum usable spans for the case is 2, the simulation results of MGDC span-1,2 show a worst performance. As a result, the equal sign in Inequality (13) is not recommended.

In Figure 5 and Figure 6, the new MGDC method gives the best performance in both mean error and standard deviation. This can be explained because the number of combinations in the new MGDC is higher than those of the other methods. In Figure 6, since the residual frequency is equal to $\frac{1}{4T_{coh}}$, the number of usable spans in MGDC method should be 1 as analyzed in Section 2.2.3. Thus, MGDC and CDC are congruent. While the MGDC method cannot improve the performance of estimation in comparison with CDC, the new MGDC reaches this goal with the best mean error and standard deviation.

In both Figure 5 and Figure 6, the performance of Kay’s method reduces rapidly when the $C/N_{0}<40$ dB-Hz. This is compatible with the analysis in Section 2.2 about the nonlinear effect of argument function on Kay’s method. Thus, in the rest of this part, which focuses on weak signals, the method will not be considered.

In the case of weak signals, the solutions to improve the performance of the receiver are increasing the number of combinations and/or using a longer integration time.

**Figure 7:**
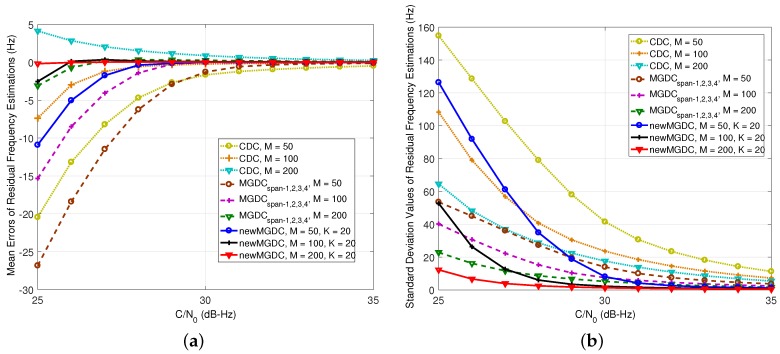
Performance of new residual frequency estimation method ($\Delta {\bar{f}}_{d}=100\;\mathrm{Hz},T_{coh}=1\;\mathrm{ms}$). (**a**) mean errors of estimations; (**b**) standard deviation of estimations.

The first solution is preferred in terms of effective implementation. In this scenario, the number of combinations for the first order differential is chosen to be 50,100,200. The number of combinations for the second order differential is K=20, this is chosen to guarantee a proper complexity. In Figure 7, because the residual is 100 Hz, even span-4 can be used for the MGDC method. It can be seen in Figure 7 that the estimations provided by the proposed method are always more accurate than those of the CDC method. Similar to Figure 5, for several low $C/N_{0}$ signals, although the traditional MGDC gives smaller estimation variances, its mean errors are larger than those of the proposed method. When the number of the second order differential is up to 200, the new MGDC gives the best performance.

**Figure 8:**
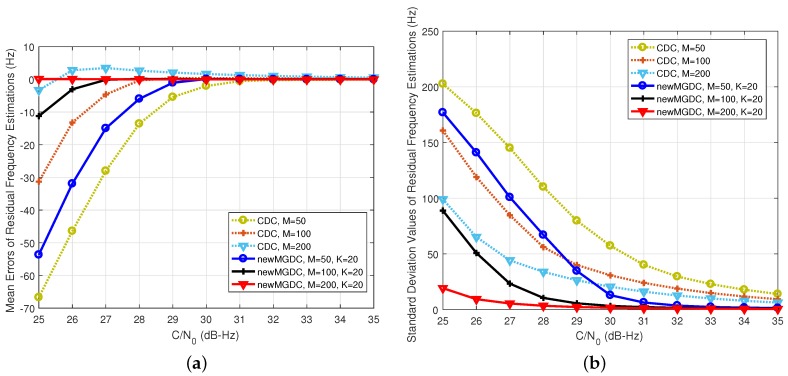
Performance of new residual frequency estimation method ($\Delta {\bar{f}}_{d}=250\;\mathrm{Hz},T_{coh}=1\;\mathrm{ms}$). (**a**) mean errors of estimations; (**b**) standard deviation of estimations.

In Figure 8, when the residual is 250 Hz, only span-1, which makes MGDC coincide with CDC, is usable. The results have the same evolution with Figure 6, where the performance of the proposed method is much better than the others. It can be seen from Figure 7 and Figure 8 that the proposed method can provide an accurate estimation even in the case of low $C/N_{0}$. The estimation error is about tens of Hertz, which is suitable to pass into a PLL.

In general, with the same number of used correlation values, the new MGDC is much more accurate than CDC. In comparison with MGDC, the improvement of the new MGDC depends on the number of combinations and the power of signal. However, it should be noted that, in order to apply MGDC, a greater frequency search space is required. It leads to the increase of computational load. This issue will be discussed more in Section 5.2.

**Figure 9:**
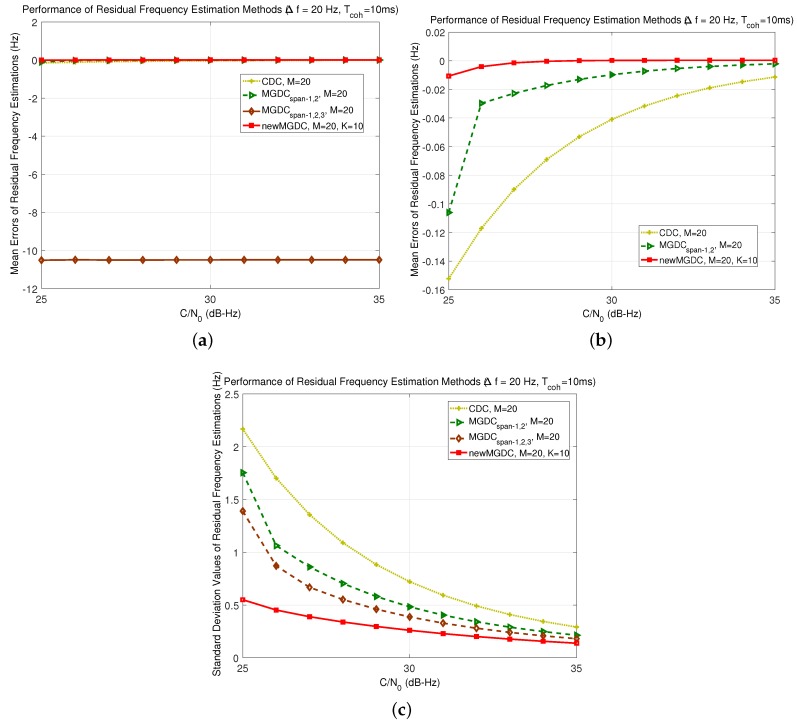
Performance of new residual frequency estimation method ($\Delta {\bar{f}}_{d}=20\;\mathrm{Hz},T_{coh}=10\;\mathrm{ms}$, M=20). (**a**) mean errors of estimations; (**b**) zoomed view of (**a**); (**c**) standard deviation of estimations.

For the second solution, which uses longer integration time, from Inequality (11), the amount of frequency error at the input of estimation will be much smaller. In Figure 9, the integration time $T_{coh}=10\mathrm{ms}$; then, the residual frequency must be smaller than 25Hz. To avoid the equal sign in Inequality (13), and possibly to compare the performance with the MGDC method, we chose $▵{\bar{f}}_{d}=20\mathrm{Hz}$. The estimation results were shown in Figure 9. Since $▵{\bar{f}}_{d}=20\mathrm{Hz}$, the maximum number of used span is 2. Thus, MGDC${}_{span-1,2}$ gives a better performance than CDC does, while the MGDC${}_{span-1,2,3}$ gives the highest error (more than 10 Hz, which is half of input error). At the same time, the new MGDC standard deviation of estimation error is the best and smaller than 1 Hz for all of the investigated signals.

### 5.2. The Computation Complexity

This part considers the computation complexity of the above methods. For Kay’s method and CDC method, their complexity can be found easily from (5) and (6). The summary of computation complexity is shown in the Table 1. The calculation for the complexity of the proposed method is described below. Since the number of correlation values is equal to *M*, the numbers of multiplications (α) and summations (β) are:

(36) $$\begin{array}{ccc} \alpha & = & \sum_{k=1}^{K}\left(M-k\right)+K-1\\ & = & K\left(M+\frac{1}{2}\right)-\frac{K^{2}}{2}-1 \end{array}$$

(37) $$\begin{array}{ccc} \beta & = & \alpha -1\\ & = & K\left(M+\frac{1}{2}\right)-\frac{K^{2}}{2}-2 \end{array}$$

The complexity of traditional MGDC can be derived in a similar manner.

**Figure 10:**
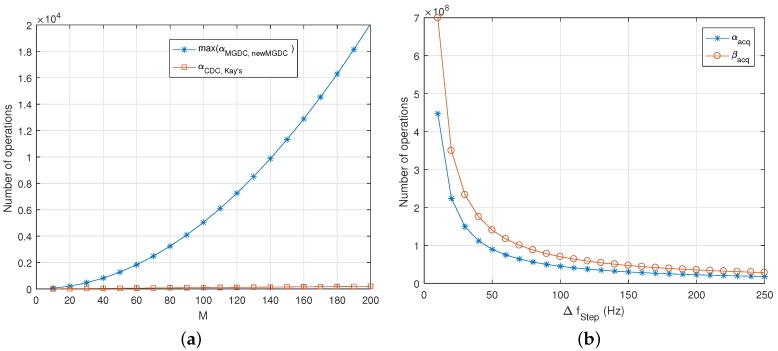
(**a**) maximum number of operations of the new MGDC method; (**b**) computational load of acquisition stage (or batch processor) at each interval using Fast Fourier Transform (FFT), $F_{s}=4$ MHz, $T_{coh}=1$ ms.

In MGDC and the new MGDC method, since K≤M−1, the number of operations is maximum when K=M−1. Figure 10a shows the maximum number of multiplications of the proposed method, when *M* is from 1 to 200, and the number of multiplications of the CDC and Kay’s method. Obviously, the complexity of the proposed method is the highest and it increases when the number of combinations grows up. However, it allows decreasing the search space in the batch processor of open-loops, and acquisition stage of closed-loops architecture, therefore reducing the overall computation burden. In more detail, from Inequality (11), the maximum frequency step is:

(38) $$\begin{array}{c} \Delta f_{Step}=\frac{1}{2T_{coh}} \end{array}$$

In the case of high dynamic users, the Doppler frequency is around ±10 kHz. Thus, the minimum size of frequency search space is:

(39) $$\begin{array}{ccc} N_{F} & = & \frac{10000\times 2}{\Delta f_{Step}}+1\\ & = & 40\times 10^{3}\times T_{coh}+1 \end{array}$$

Obviously, the longer the integration time, the greater the size of frequency search space.

The load of calculations for the correlators using Fast Fourier Transform (FFT) at each interval is given by [27]:

(40) $$\begin{array}{cc} \alpha_{acq} & =N_{F}\left(4N\log_{2}4N\right)+2N\log_{2}4N \end{array}$$

(41) $$\begin{array}{cc} \beta_{acq} & =N_{F}\left(6N\log_{2}6N\right)+3N\log_{2}6N \end{array}$$

where $N=T_{coh}F_{s}$ is the number of samples for one integration interval.

From Equation (40), the change of computational load of acquisition stage (or batch processor) versus the Doppler step is shown in Figure 10b. Comparing with the maximum number of operations of the new MGDC method in Figure 10a, it can be seen that the computational burden of acquisition stage is much higher than that of the frequency estimators. Therefore, to save on the computational cost, reducing the search space is more effective than choosing a low cost frequency estimator.

To apply the MGDC method, the size of search space must be increased depending on the number of spans we want to use. For example, using up to MGDC span-2 requires the double density of search space. Similarly, MGDC span-3, 4, 5 requires the frequency grid to be three, four, or five times denser than CDC and the new MGDC do. In more detail, assume that the sampling frequency is 4 MHz and the integration time $T_{coh}=1$ ms; then, N=4000 (samples) and the minimum number of searched frequencies $N_{F}=41$. At each interval, the number of multiplications and summations are $4.8042\times 10^{6}$ and $7.5082\times 10^{6}$, respectively. For each added frequency in the search space, the number of calculations increases $5.2383\times 10^{5}$. This number will be much greater if a longer integration time and/or a greater sampling frequency are used. Considering Equations (36) and (37), if M≤200 and K≤M−1, the load of calculations of the new MGDC is much smaller than that of correlators in the acquisition stage or batch processors.

Another considerable example is the particular cases in Figure 7 and Figure 8, in which the standard deviation of estimation error ($▵\hat{f}$) is about 50 Hz at $C/N_{0}=26$ dB-Hz. The methods and their parameters are summarized in Table 2. It can be observed that, while the difference between the complexity of CDC and MGDC is not much, the number of multiplications and summations of new MGDC is so high. However, the key difference here is the frequency error $\Delta {\bar{f}}_{d}$ at the input of each method. In normal cases, if the input frequency error is $\Delta {\bar{f}}_{d}$, from Equation (39), the maximum frequency step in search space is: $\Delta f_{Step}=\left|2\Delta {\bar{f}}_{d}\right|$. Therefore, the minimum value of $N_{F}$ must be:

(42) $$\begin{array}{c} \min\left(N_{F}\right)=\left\lceil \frac{10000}{\Delta {\bar{f}}_{d}}\right\rceil +1 \end{array}$$

Hence, in the considering condition ($C/N_{0}=26$ dB-Hz, $var(▵\hat{f})=50$ Hz), the acquisition stage requires a minimum frequency space size of 101 for CDC and MGDC${}_{span-1,2,3,4}$, and 41 for the new MGDC. Thus, the load of calculation in the acquisition stage can be reduced to less than a half if the new MGDC is then applied.

## 6. Conclusions

In this paper, a new method to estimate the residual frequency for GNSS signal processing is proposed. This new method gives a higher accuracy than the traditional method, such as: Kay’s, CDC, and the MGDC method. Moreover, although based on the idea of MGDC, it is not constrained by the amount of residual frequency input as the MGDC method. The complexity of the proposed estimation method is higher than the others; however, it reduces considerably the complexity of the acquisition stage. The proposed method is suitable to be adopted to the frequency estimator in open-loop tracking architecture, or applied in closed-loop to reduce the transient time from acquisition to tracking.

In future works, the effect of data transition and Doppler drift will be considered. A full open-loop architecture, which uses the new MGDC method to estimate frequency, is going to analyze in both theoretical scenarios and simulation.

## Figures and Tables

**Table 1:** The computation complexity of different methods.

Method	Arg	α	β
classical Kay	M−1	M−1	M−1
Conventional Differential Combination (CDC)	1	M−1	M−1
Modified Generalized Differential Combination (MGDC)	*K*	$\sum_{k=1}^{K}\left(M-k\right)+K$	$\sum_{k=1}^{K}\left(M-k\right)+K-1$
new MGDC	1	$\sum_{k=1}^{K}\left(M-k\right)+K-1$	$\sum_{k=1}^{K}\left(M-k\right)+K-2$

**Table 2:** Comparing the computation complexity in the same estimation accuracy.

Method	CDC $\Delta f_{d}=100$ Hz, M=200	MGDC${}_{\mathrm{span}-1,2,3,4}$ $\Delta f_{d}=100$ Hz, M=50	new MGDC $\Delta f_{d}=250$ Hz, M=100,K=20
arg	1	4	1
$\alpha_{freq.estimation}$	199	194	1909
$\beta_{freq.estimation}$	199	193	1908
$N_{F}$	101	101	41
$\alpha_{acq}$	$2.2680\times 10^{7}$	$2.2680\times 10^{7}$	$9.273\times 10^{6}$
$\beta_{acq}$	$3.5446\times 10^{7}$	$3.5446\times 10^{7}$	$1.4493\times 10^{7}$
